# Whole-Genome Sequence of a Freshwater Aerobic Anoxygenic Phototroph, *Porphyrobacter* sp. Strain AAP82, Isolated from the Huguangyan Maar Lake in Southern China

**DOI:** 10.1128/genomeA.00072-13

**Published:** 2013-03-07

**Authors:** Xiuling Li, Michal Koblížek, Fuying Feng, Yunxu Li, Jichang Jian, Yonghui Zeng

**Affiliations:** a Shangdong Provincial Key Laboratory of Water and Soil Conservation & Environmental Protection, College of Resources and Environment, Linyi University, Linyi, China; b Institute of Microbiology CAS, Opatovický mlýn, Třeboň, Czech Republic; c Institute for Applied & Environmental Microbiology, Life Sciences College, Inner Mongolia Agricultural University, Huhhot, China; d Guangdong Provincial Key Laboratory of Pathogenic Biology and Epidemiology for Aquatic Economic Animals, Guangdong Ocean University, Zhanjiang, China

## Abstract

The *Porphyrobacter* genus (of the class *Alphaproteobacteria*) contains aerobic anoxygenic phototrophic species. Here we report a draft genome sequence of a freshwater bacterium, *Porphyrobacter* sp. strain AAP82. It contains a 38-kb-long photosynthesis gene cluster, but carbon-fixation genes are absent. The presence of respiratory enzymes, tricarboxylic acid (TCA) cycle, and the Entner-Doudoroff pathway demonstrates its aerobic photoorganotrophic character.

## GENOME ANNOUNCEMENT

Aerobic anoxygenic phototrophs (AAPs) represent an important fraction of bacterioplankton in the oceans (1, 2) and in freshwater systems (3–5). Contrasting with successful efforts to accumulate data on the ecology of AAPs, efforts toward AAP isolation and sequencing of their full genomes are still insufficient, limiting our understanding of their metabolic capabilities. The *Porphyrobacter* genus belongs to the family *Sphingomonadaceae* of the *Alphaproteobacteria* and is one of few formally named exclusively AAP genera. A number of species have been described from this genus (6–11), but their genome sequences are unavailable. In December 2011, we isolated a yellow-pigmented *Porphyrobacter* sp. strain AAP82 from the surface water of the Huguangyan Maar Lake in southern China and sequenced its genome with the aim to assess its metabolic character and photosynthesis gene composition.

*Porphyrobacter* sp. AAP82 was grown in liquid R2A medium and DNA was extracted using a genomic DNA purification kit. A 300-bp DNA library for Illumina sequencing technology was constructed. An Illumina HiSeq 2000 platform was employed for sequencing. Approximately 11.2 Gb raw data of 101-bp-long pair-end reads were generated. A total of 43,700,870 reads were subjected to quality control and trimming on the Galaxy server (12) and then were *de novo* assembled into contigs using the Velvet program (Ver. 1.2.08) (13). Various hash lengths between 31 and 91 were tested and an optimal assembly was achieved with a k-mer size of 57. Contigs less than 300 bp long were removed from the final assembly. Genome coverage was ca. 200×. Annotation was performed with the RAST (14) and BASys (15) servers.

The draft genome consisted of 52 contigs with an N_50_ value of 116,777 bp and a total length of 2.9 Mb, containing 2,791 open reading frames (ORFs) and 44 tRNAs. The G+C content was 67.3%. One complete rRNA operon was assembled. The 16S rRNA gene sequence of *Porphyrobacter* sp. AAP82 shows 99.5% identity to that of the *Porphyrobacter donghaensis* strain SW-158 and 98.5% to *P. neustonensis* DSM 9434. A 38,225-bp-long photosynthesis gene cluster (PGC) was located in a 116,777-bp-long contig with a gene organization of *bchIDO*-ORF-*arsR*-3×ORFs*-crtCDF-bchCXYZ-pufBALM-*ORF*-tspO-bchP-pucC-bchG-ppsR-*ORF*-bchFNBHLM-lhaA-puhABC*-ORF-*acsF-puhE*. Its *pufL* and *pufM* protein sequences show 96%/93%, 93%/83%, and 91%/81% identities to those of *P. neustonensis*, *P. sanguineus*, and *P. tepidarius*, respectively.

There are no autotrophic CO_2_ fixation pathway genes in the draft genome sequence. A key gene encoding phosphofructokinase for glycolysis is absent, but a complete set of genes for the tricarboxylic acid (TCA) cycle and Entner-Doudoroff pathway are predicted. There are genes encoding urea carboxylase, propionyl-coenzyme A (CoA) carboxylase, methylcrotonyl-CoA carboxylase, biotin carboxylase, phosphoenolpyruvate carboxylase, and carbonic anhydrase. Genes encoding sulfate/sulfite/nitrite/nitrate reductase are not present. Some genes associated with the flagellar system were identified (*flaA*-ORF-*motBA-flgL-fliA*). Various proteins that are closely related to an aerobic lifestyle were identified, including NADH dehydrogenase, cytochrome *c* oxidase, succinate dehydrogenase, and catalase, which together with the presence of the PGC confirm that *Porphyrobacter* sp. AAP82 is an aerobic anoxygenic phototroph.

### Nucleotide sequence accession numbers.

This whole-genome shotgun project has been deposited at DDBJ/EMBL/GenBank under the accession number ANFX00000000. The version described in this paper is the first version, ANFX01000000.
